# Insulin Resistance and Bone Metabolism Markers in Women with Polycystic Ovary Syndrome: A Cross-Sectional Study on Females from the Islamic University Medical Center

**DOI:** 10.3390/medicina59030593

**Published:** 2023-03-16

**Authors:** Fahad Khalid Aldhafiri, Fathy Elsayed Abdelgawad, Gihan Mohamed Mohamed Bakri, Tamer Saber

**Affiliations:** 1 Public Health Department, College of Applied Medical Sciences, Majmaah University, Al Majmaah 11952, Saudi Arabia; 2 Medical Biochemistry Department, Faculty of Medicine, Al-Azhar University, Cairo 11651, Egypt; 3 Chemistry Department, Faculty of Science, Islamic University of Madinah, Madinah 42351, Saudi Arabia; 4 Female Section, University Medical Center, Islamic University of Madinah, Madinah 42351, Saudi Arabia; 5 Internal Medicine Department, Faculty of Medicine, Zagazig University, Zagazig 44511, Egypt

**Keywords:** insulin resistance, bone metabolism, PCOS, anthropometric measures, biochemical parameters, endocrine activity, clinical parameters

## Abstract

*Background and Objectives*: polycystic ovarian syndrome (PCOS) prevails in females in the 18–40-year-old age group and varies from 5–20% depending on the demographic and diagnostic standards. It is unknown how long passes between the onset of a specific symptom and the appearance of the disease. The three most significant characteristics of PCOS include irregular menstruation, a polycystic ovarian shape found by pelvic ultrasound, and hyperandrogenism, which could possibly delay menarche. This study’s objective was to assess insulin resistance and bone bio-markers’ metabolism-involved characteristics of females with PCOS. *Materials and Methods*: We present a cross-sectional study carried out on 100 female patients suffering from PCOS and 100 healthy female subjects as a control living in Saudi Arabia in the Al-Madinah Al-Munawara Region between May 2021 and March 2022. The age of the studied groups ranges from 20–40 years, and patients were categorized into three groups; group I (control, n = 100), group IIa (overweight or obese females with PCOS, n = 70), and group IIb (non-obese females with PCOS, n = 30). The diagnosis of PCOS was carried out as per Rotterdam criteria as recommended for adolescent and adult subjects. All the groups were subjected to physical examination, and anthropometric measures, biochemical parameters, endocrine activity, and clinical parameters were determined. The data obtained were computerized and analyzed statistically using the SPSS program for range, mean, and standard deviation. ANOVA test with post hoc Tukey test was applied to assess the pattern and variation among the test and control groups. *Results*: In the present study, age, waist circumstances, systolic blood pressure, and diastolic blood pressure were reported enhanced in the PCOS over the control group. Additionally, anthropometric measures were reported slightly upregulated in group IIa over group IIb (*p* < 0.001). Biochemical parameters including glucose, insulin incidence, and lipids were reported higher in the PCOS over the control group, where group IIa showed slightly increased values compared to group IIb (*p* < 0.001). On the contrary, PTH, Ca^+2^, and 25(OH)D levels were reported lower in the PCOS over the control group. However, in the control groups, a slight variation was reported as higher in group IIa compared to group II. In the study, PTH and 25(OH)D were found associated with bone metabolism; a lower level of PTH and 25 (OH) D is linked with a decline in bone density. *Conclusions*: Lower serum levels of PINP and osteocalcin along with the 25(OH)D were associated with the PCOS compared to the control group, imposing a higher risk of the syndrome. On the contrary, an elevated level of NTx in groups IIa and IIb over the control group was associated with insulin resistance and bone metabolism.

## 1. Introduction

A complicated endocrine and metabolic disorder called polycystic ovarian syndrome (PCOS) is associated with chronic anovulation/oligomenorrhea, hyperandrogenism, and insulin resistance [1]. The European Society for Human Reproduction and Embryology and the American Society for Reproductive Medicine (ESHRE/ASRM) established the Rotterdam guidelines for PCOS in 2003 [2,3]. The diagnosis of PCOS is a challenging procedure that necessitates ruling out other probable reasons, notably hyperandrogenism and menstrual irregularities (hyperprolactinemia, non-classical congenital adrenal 21-hydroxylase deficiency, thyroid disorders, androgen-secreting malignancy, and Cushing’s disorder). When at least two of the following factors for polycystic ovary syndrome are present, namely oligomenorrhea or anovulatory cycles with anomalous menstrual cycle, increased levels of circulating androgens or clinical manifestations of androgen excess, and ultrasound evidence of polycystic ovary syndrome, the disorder can be scientifically diagnosed and defined. Nearly 5–10% of females of reproductive age develop PCOS, which is accompanied with endocrine abnormalities. PCOS varies in the population globally [4,5,6]. Moreover, the incidence of PCOS differs among populations based on the testing parameters used, with the incidence rate as per the Rotterdam criteria being approximately 2–3 times higher than as per those based on the National Institutes of Health (NIH) criteria [7,8,9,10,11,12].

Insulin resistance (IR) and PCOS are closely linked in female populations across the world. Because insulin’s physiological functions such as carbohydrate intake and metabolism, glucose synthesis, and lipid metabolism are no longer as effective, increased insulin levels are essential to achieve adequate metabolism function in individuals with insulin resistance. When the pancreatic beta cells are physiologically normal, there is an increased level of circulating insulin whenever IR is present [13]. Insulin promotes tyrosine phosphorylation on tyrosine residues and stimulates a cell’s intrinsic kinase after interacting with a receptor on the cell surface [14,15]. As per studies, diminished receptor adhesion in insulin signaling promotes insulin sensitivity, decreasing in PCOS females. The primary contributor to a reduction in insulin sensitivity is the serine phosphorylation of the insulin receptor and IRS-1 by intracellular serine kinase. Consequently, PCOS females exhibit decreased insulin mediated PI3K activation and resistance to insulin’s metabolic changes [16]. Obesity is still a crucial variable in PCOS women’s condition, although the post-receptor mechanism disruption is associated with insulin-resistant and lean/normal-weight in such females [17,18]. PCOS in females is connected to hyperinsulinemia and hyperandrogenemia, in which the ovary preserves its sensitivity to insulin activity and, as a response, produces androgen in addition to systemic insulin resistance [19,20].

Furthermore, research has shown that females with PCOS as per predefined criteria for the multiple sclerosis (MS) often have an increased prevalence of hypertension, dyslipidemia, and abdominal obesity [21]. For a clinical diagnosis of the metabolic syndrome, central adiposity should be linked to at least two of the following pathophysiological symptoms: hypertension, elevated triglyceride levels, decreased high-density lipoprotein cholesterol (HDL-C) levels, or a rise in fasting blood sugar levels [22]. As per studies, several PCOS females are more resistant to insulin than control-group females despite meeting the same age and body mass index (BMI) criteria. Additionally, compensatory hyperinsulinemia remains involved prominently with IR in PCOS females [23]. Obesity remains one of key causes for insulin resistance in PCOS females; however, recent research has demonstrated that this condition is independent of body weight [24]. Overall, 3% of the human genome is subject to the control of the vitamin D receptor gene that also controls blood pressure and genes involved in lipid and glucose metabolism [25,26]. Females having PCOS who do have metabolic syndrome also depend on vitamin D, and it has been demonstrated that serum vitamin D levels in the blood enhance the risk of MS [27].

The hormones in IR, hyperinsulinemia, and obesity have an impact on calcium homeostasis. Vitamin D serum levels in obese people and PCOS women are low, whereas parathyroid hormone (PTH) concentrations are higher [28,29,30]. Earlier studies have demonstrated a relationship between PCOS as well as certain bone health factors, most notably a decrease in osteocalcin, which is a marker of bone development, as well as decrease in spinal and femoral bone mass in PCOS individuals with BMIs ≤ 27 kg/m^2^ [31,32]. An impact of androgens on females is not completely explored, even though estrogen is essential for growth and maintaining bone density in females. The ovary as well as other glandular tissue is believed to be where androgens transform into estrogens, which then attach to estrogen receptors in the target tissues. Androgens have an influence on bone metabolism in this manner [33]. Therefore, the hyperandrogenism brought on by PCOS in women’s ovarian and adrenal glands could have an influence on bone turnover and bone mineral density (BMD). On the other hand, menstruation disruption at this critical period may have a similar effect since maximal bone mass is achieved between the late teenage years to the mid-thirties [34]. The prospective risk for osteoporosis among young women with PCOS because of their irregular periods of menstruation and amenorrhea is not yet established. BMD alterations, BMD increases, or BMD losses have not really been observed in PCOS affected women either [35,36]; hence, it is unknown whether BMD changes occur with PCOS [37].

## 2. Aim of the Study

The rationale of present investigation is to explore and characterize IR, biochemical features of bone metabolism, and association with metabolic characteristics in females with PCOS. The study also examines and describes IR, biochemical markers of bone metabolism, and association with metabolic parameters in females with PCOS.

## 3. Subjects and Methods

A maximum of 200 participants, namely 100 clinically diagnosed PCOS individuals and 100 control participants, were enrolled in the cross-sectional study. The number of individuals participating in this study was calculated according to the equation for calculating sample size, which is as follows: *=n*/(1 + (*n* − 1)/*P*) where A is the adjusted sample size, n is the sample size, and P is the population size.

Between May 2021 and March 2022, the study was conducted at Al-Madinah Al-Munawara, Saudi Arabia. The participants who signed consent were between the ages of 20 and 40. During follow-up at the obstetrics and gynecology clinic at the Islamic University Medical Center, subjects were chosen from outpatient clinics. According to the diagnosis, the included participants were separated into two groups, with group II being further divided into group IIa and group IIb.

1. Group I included 100 healthy female patients as controls;
2. Group IIa included 70 overweight or obese female patients diagnosed as having polycystic ovary syndrome and with BMI above 25 kg/m^2^;
3. Group IIb included 30 non-obese female patients diagnosed as having polycystic ovary syndrome and with BMI less than 25 kg/m^2^. All study subjects were subjected to physical, anthropometric, and laboratory examinations.

### 3.1. Exclusion Criteria

All 200 enrolled individuals in this instance met the predetermined inclusion criteria. Subjects in the study group, who were all from twenty to forty years of age, demonstrated PCOS symptoms in compliance with the Rotterdam diagnoses. The additional diagnoses of oligo-anovulation, such as hyperprolactinemia, Cushing’s disease, untreated hypothyroidism, congenital adrenal hyperplasia, and adrenal tumors, were also excluded. Participants who reported using androgens, valproic acid, cyclosporine, diazoxide, or minoxidil; having taken oral contraceptives, metformin, thiazolidinediones, or spironolactone for longer than the previous three months; or who were pregnant were also excluded from the study. Before a clinical diagnosis of PCOS, participants who had diabetes mellitus (pre-existing) were excluded from the research.

### 3.2. Physical Examination and Anthropometric Measures

The physical examination included a chest, abdominal, and neurological examination with stress on blood pressure measurement. The blood pressure was measured and recorded in the enrolled participants using standard laboratory conditions. Both systolic and diastolic pressures were checked and recorded twice using an automated blood pressure measurement instrument and the appropriate-sized cuff (bladder within the cuff must encircle 80% of the arm), with measurements separated by two minutes. Anthropometric parameters were determined as per the standard protocols. The body weight of all the participants in both the study and control groups was determined to the nearest 200 gm. BMI was determined with a ratio of weight (kg) over height squared (m^2^). WC was determined to the nearest 0.5 cm at the end of expiration at the midpoint between the top of the iliac crest and the lowest rib in an upward orientation. Patients with PCOS were diagnosed according to the Rotterdam criteria [2].

The main clinical symptoms and symptoms of PCOS are mentioned below, although not every PCOS patient will encounter these: regular missing periods, obesity increase particularly around the waist, excessive facial hair development (hirsutism), persistent acne (generally resistant to conventional therapy), and greasy skin are the chief clinical signs linked to PCOS. The diagnosis of PCOS also points to loss of hair on the scalp, difficulties conceiving or having children, significant dark skin spots (acanthosis nigricans) and skin tags, moderate to severe pelvic pain, mood swings, anxiety, and depression.

Here, in present study, for all the registered participants, the Rotterdam criteria were applied for diagnosing PCOS, including two out of the three features in the revised criteria:

1. Oligomenorrhea (irregular menstrual periods) or amenorrhea (absence of menstrual periods);
2. Hyperandrogenism (based on clinical signs in the body) and/or biochemical signs (hormone levels in the blood);
3. Polycystic ovaries (on the ultrasound): Polycystic ovaries are described on an ultrasound scan as the “presence of 12 or more follicles in one or both ovaries measuring 2–9 mm in diameter, and/or increased ovarian volume (>10 mL)” [38].

### 3.3. Laboratory Measurements

After a 10–12 h overnight fasting, fresh blood samples were collected from the study and control groups in the morning. The blood was drawn from the antecubital vein between the second and the fifth days of the cycle, during the early follicular phase. Obtained blood specimens were centrifuged to collect the serum after 20 min of clotting time. Using a standard biochemical laboratory diagnostic protocol, the serum concentrations of calcium, phosphorus, total alkaline phosphatase (ALP), fasting glucose (FG), total cholesterol (TC), total triglycerides (TG), high-density lipoprotein cholesterol (HDL-cholesterol) and low-density lipoprotein cholesterol (LDL-cholesterol), calcium, phosphorus, and total alkaline phosphatase (ALP) were calculated with an automated Siemens Dimension Clinical Chemistry System [39,40,41,42].

The Vitros 3600 (Ortho–clinical Diagnostic, Johnson, and Johnson Co., Colorado Springs, CO, USA) immunodiagnostic system’s Reagent Pack and Vitros calibrators were made to produce the result of estrogen, follicle-stimulating hormone (FSH), luteinizing hormone (LH), prolactin (PRL), total testosterone, 25-hydroxyvitamin D (25(OH)D), and intact parathyroid hormone (iPTH) [43,44,45,46,47,48,49,50,51]. Chemiluminescent micro particle immunoassay (CMIA) was used for the quantitative determination of architect dehydroepiandrosterone sulfate (DHEA-S), fasting insulin (FI), and sex hormone-binding globulin (SHBG) [52,53,54]. Further, intact human osteocalcin was determined using ELISA, 96-well plates, and a micro plate reader [55]. Patients were instructed to submit a second-morning void urine sample for quantitative NTx assessment using the Vitros NTx Reagent Pack and Vitros NTx calibrators on the Vitros immunodiagnostic instrument. Bone collagen was quantified in the recent research as nanomoles of bone collagen equivalent per liter (nM BCE) [56,57]. Using the continuity formula, urine dilution was adjusted utilizing urine creatinine analysis and nanomoles of bone collagen equivalent per liters per millimole creatinine [58].

$$\mathrm{NTx}\;\left(\mathrm{nMBCE}/\mathrm{mMcreatinine}\right)=\;\mathrm{NTx}\;\left(\mathrm{nMBCE}\right)/\mathrm{Urinary}\;\mathrm{creatinine}\;\left(\mathrm{mM}\right)$$

Similarly, non-HDL cholesterol was calculated using following equation [59].

Non−HDL = TC –HDL cholesterol

Additionally, the Vermeulen formula was used for free testosterone calculation [60].

The studies also included dual-energy X-ray absorptiometry (DXA scan), the qualitative insulin sensitivity check index (QUICKI), and the homeostasis model evaluation of insulin resistance (HOMA-IR). Using fasting insulin and fasting glucose levels, HOMA-IR determined insulin resistance using the following formula: [insulin (uU/mL)] [fasting glucose (mg/dL)]. HOMA-IR higher than 2.5 was employed to define insulin resistance [61].

Here, in the present study, both fasting insulin (uU/mL) and glucose (mg/dL or mmol/L) levels from blood samples were determined using QUICKI formula. The QUICKI formula for insulin and glucose level used for insulin resistance/sensitivity is shown below [62].

$$1/\log\left(\mathrm{Fasting}\;\mathrm{Insulin}\right)+\;\log\;\left(\mathrm{Fasting}\;\mathrm{Glucose}\right)$$

Bone densitometry, also known as scanning dual-energy X-ray absorptiometry (DXA), was employed to examine and estimate bone-loss measurement. DXA provides a consistent method for determining bone mineral density (BMD) [63,64].

### 3.4. Statistical Analysis

Here, in present study, Statistical Package for Social Science (SPPS) version 27.0 was used for the analysis of collected data, anthropometric measures, and laboratory measurements. The analyzed data from the present study are summarized in tables as range, mean, and standard deviations (SD). Further, analysis of variance (ANOVA) along with post hoc Tukey test was performed to examine the difference between the study and control groups. Pearson correlation coefficient was used to analyze two quantitative variables. Statistical significance was detected when the *p*-value was equal to or less than 0.05, and high significance was detected when the *p*-value was less than 0.001.

## 4. Results

Table 1, Table 2, Table 3, Table 4 and Table 5 demonstrate both the biochemical and clinical parameters of the study groups categorized in accordance with the polycystic ovary syndrome criteria. There was no statistically significant difference in age, SBP, DBP, TG, FSH, PRL, PTH, ALP, NTx, or BMI Z or T score between the groups in investigation. Nevertheless, there was a statistically significant difference in the WC, BMI, FG, and FI between groups I and IIa. The biochemical parameters showed a statistically significant difference in HOMA, QICKI, cholesterol, HDL, non-HDL, LH, estrogen, SHBG, female total testosterone, female free testosterone, female testosterone percent, DHEAS, total Ca, pH, 25(OH) D, osteocalcin, and PINP. HOMA, QICKI, cholesterol, HDL, LDL, LH, estrogen, SHBG, female total testosterone, female free testosterone, female testosterone percent, DHEAS, albumin-corrected total Ca, P, 25(OH)D, osteocalcin, and PINP were significantly different between the groups I and IIb. In terms of cholesterol, LDL, and non-HDL, there was no statistically significant distinction between group I and group IIb. Between groups IIa and IIb, there was a statistically significant difference in WC, BMI, cholesterol, LDL, non-HDL, SHBG, DHEAS, 25(OH)D, osteocalcin, and PINP. In terms of FG, FI, HOMA, QICKI, HDL, LH, estrogen, female total testosterone, female free testosterone, female testosterone percent, Ca, P, osteocalcin, and PINP, there was a lack of significant difference between groups IIa and IIb.

In Table 6, there is a statistically significance positive correlation between NTx and QICKI and a statistically significance negative correlation between NTx and BMI, insulin, HOMA IR, cholesterol, and female testosterone. There was a statistically significance positive correlation between osteocalcin and parathyroid, ALP, and 25(OH)D and a statistically significance negative correlation between osteocalcin and prolactin. Finally, there was a statistically significance positive correlation between PINP and WC and testosterone.

## 5. Discussion

**Figure 1:**
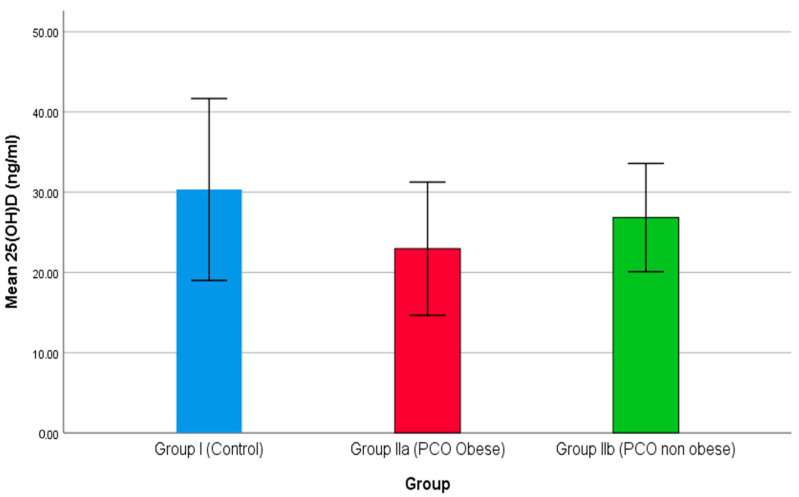
The comparison of 25(OH)D between studied groups.

In this cross-sectional study, 200 females attending the Islamic University Medical Centre were grouped into group I, including 100 healthy females; group IIa 70, including overweight or obese females with PCOS; and group IIb, including 30 non-obese females with PCOS. Figure 1 demonstrates the comparison of mean 25(OH)D levels among the three groups of participants. As the results show in Figure 1, group II (obese females with PCOS) reported a minimum level of serum means of 25(OH)D ng/mL compared to the other two groups i.e., IIb (non-obese with PCOS) and control (group I). A higher level of mean 25(OH)D ng/mL was reported in the control group (group I). Previously, Lin and Wu (2015) demonstrated the role of vitamin D in polycystic ovary syndrome [65]. Thomson et al. (2012) determined the level of 25(OH)D in healthy and PCOS patients. A reduced level, i.e., less than 20 ng/mL of 25(OH)D triggers a higher prevalence of PCOS cases (67–85%) [66]. Additionally, a low level of 25(OH)D is also involved with IR in females with PCOS [67,68].

**Figure 2:**
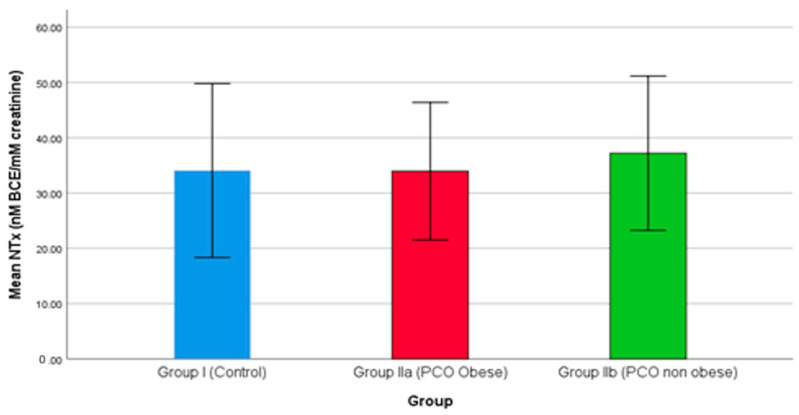
The comparison of NTx between studied groups.

Bone turnover is directly associated with the rate of osteoporosis, and the role of 25(OH)D is pivotal in bone density. In the present study, serum levels of NTx were examined in all three groups and were reported higher in PCOS patients compared to the control. Interestingly, as the results show in Figure 2, group IIb, i.e., non-obese females with PCOS, reported higher serum levels of NTx than group IIa, i.e., obese females with PCOS. Previously, Iba et al. (2008) demonstrated a higher risk of bone turnover in patients with an elevated level of NTx. NTx is a potential biomarker for bone density and bone re-absorption. The serum NTx level changes significantly after menopause, and a declining level of 25(OH)D poses further risk of PCOS [69]. The serum NTx level is not directly associated with PCOS; however, elevated plasma levels of (25(OH)D play a pivotal role in minimizing the risk of PCOS.

**Figure 3:**
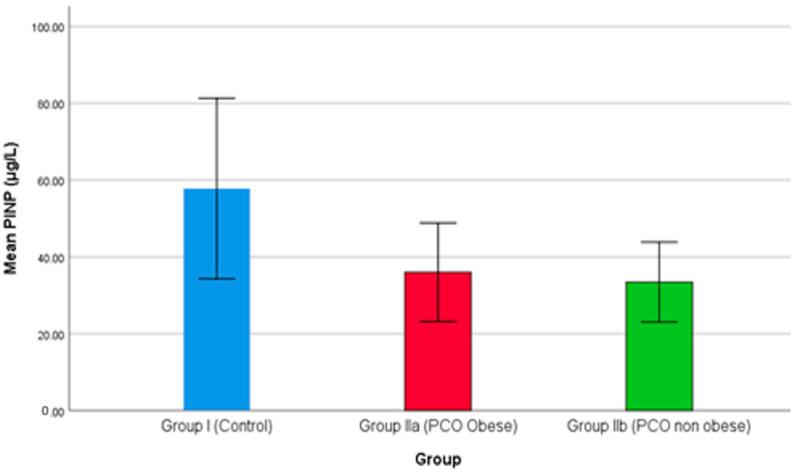
The comparison of PINP between studied groups.

Procollagen type I N propeptide (PINP) serum levels were compared among the three groups and are demonstrated in Figure 3. As the results show in Figure 3, both groups IIa and IIb reported a declining level of PINP. In the study, the PINP level was higher in the control group than the PCOS groups, while group IIa (obese females with PCOS) showed significantly higher levels than group IIb (non-obese females with PCOS). PINP is the marker for bone formation, and as the results show in Figure 3, both groups IIa and IIb reported a sharp decline in serum level; hence, bone formation remains hampered. Lingaiah et al. (2017), in a multicenter study, demonstrated that a decreased level of PINP is associated with PCOS [70]. Additionally, the PINP level is also associated with 25(OH)D level and with risk factors of hyperandrogenism, hyperinsulinemia, and obesity [71]. 

**Figure 4:**
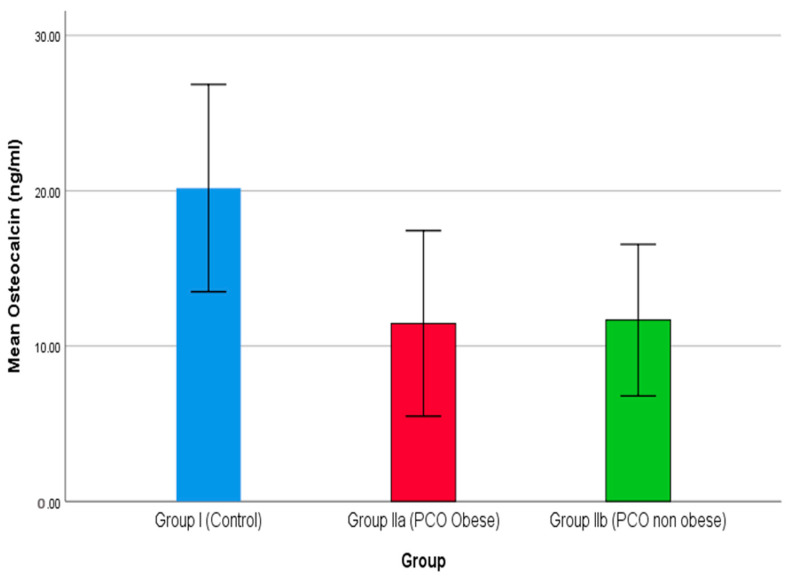
The comparison of osteocalcin between studied groups.

PCOS poses a negative impact on bone health, where obesity is a crucial risk factor associated with 25(OH)D [72]. PTH indirectly enhances vitamin D via an increased level of Ca^+2^. Additionally, an increase in PTH level also enhances the PINP level [73]. In the present study, a declining level of PINP reported in the two groups, i.e., IIa and IIb, indicates a higher risk of PCOS. Similarly, comparison of osteocalcin among the three groups is shown in Figure 4, where groups IIa and IIb demonstrate a declining level of osteocalcin compared to group I (control). Additionally, group IIa reported the lowest level of osteocalcin, which demonstrates that obesity is a key cause of PCOS. Serum osteocalcin is a key biomarker for osteoporosis. On the contrary, Singh et al. (2015) reported a higher level of serum osteocalcin in a case-control study where patients showed a sharp rise in osteocalcin level over the control [74]. Vs et al. (2013) report a negative correlation between bone mineral density and osteocalcin level [75].

In the present study, physical examination, and anthropometric measures among all the three groups are summarized in Table 1. As the data show in Table 1, five variables including age, waist circumference, body mass index, systolic blood pressure, and diastolic blood pressure were examined. Among these variables in different groups, the BMI findings of group IIa were significant, while the other two groups were highly significant. Similarly, WC findings demonstrated significant outcomes in the post hoc test. Chitme et al. (2017) investigated an association between physical examination and anthropometric measures and the risk of PCOS. A hospital-based case-controlled study with 132 patients along with control reported a mean age with a higher incidence of 29.74 ± 3.32 years. Additionally, BMI and WC in cases and controls were reported at 28.2 ± 6.08, 97.44 ± 15.11 cm, and 109.22 ± 17.39 cm, respectively [76]. Lim et al. (2012) and Borruel et al. (2013) in their studies also reported a similar pattern of physical examination and anthropometric measures [77,78]. The comparison of other variables remains non-significant. In Table 2, biochemical parameters, glucose, and insulin indices are summarized. As the results show in Table 2, fasting glucose, fasting insulin, HOMA IR, and QICKI were determined for group I (control), IIa (obese females with PCOS), and IIb (non-obese females with PCOS). In the present findings, except for the non-obese females with PCOS, the other two groups, namely the control and obese females with PCOS, reported significance in all the variables, while group IIb’s reports were non-significant. Najem et al. (2008) demonstrated clinical and biochemical profiles in PCOS cases in a retrospective study. Here, in the present study, 10% were reported diabetic, with an elevated level of serum testosterone and serum prolactin at 26% and 31%, respectively. PCOS prevalence depends on multiple risk factors, while obesity and insulin resistance remain critical [79]. According to Mario et al. (2012), a higher risk of PCOS was reported in cases with central obesity and insulin resistance [80].

The lipid profile in the study group showed a similar pattern, as shown in Table 3. Here, in the lipid profile analysis, group IIa reported non-significance (*p* > 0.05), while the other two groups were significant (*p* < 0.05). Table 4 summarizes the association between hormones and PCOS in the study groups. Here, in the present study, the serum level of FSH and PRL showed a non-significant difference between the control group and PCOS groups (*p* > 0.05), while LH, estrogen, SHGB, female total and free testosterone, and DHEAS showed a significant difference and a highly significant difference between the control group and PCOS groups (*p* < 0.05) and (*p* < 0.001).

Vitamin D level is crucial in bone density and health in the present study, namely parameters PTH, Ca, PH, ALP and 25(OH)D, NTs, osteocalcin, and PINP. The serum level of PTH was reported as NS (*p* = 0.17), and the Ca level was highly significant (*p* < 0.008) for the control group and significant (*p* < 0.03) for group IIa while significant (*p* > 0.05) for group IIb. A similar pattern was reported for the Ph level, where it was highly significant (*p* < 0.001) for the control group and significant (*p* < 0.02) for group IIa while non-significant (*p* > 0.92) for group IIb. For the ALP, all the study groups reported serum levels that were non-significant (*p* < 0.11). In the study, levels of 25(OH)D were reported as highly significant among the three study groups (*p* < 0.001). On the contrary, NTx serum level in the study groups was reported as non-significant (*p* = 0.09). Additionally, serum levels of osteocalcin and PINP were reported as highly significant (*p* < 0.001) for the control group (group I) and group IIa and non-significant (*p* = 0.95 and 0.43) for group IIb, respectively. Table 6 summarizes the Pearson correlation coefficient where female testosterone and 25(OH)D are associated with a lower level of osteocalcin. Sam et al. (2015) examined the level of hormones, namely 25(OH)D, PTH, and testosterone, in the increase of PCOS cases [81]. Previously, Glintborg et al. (2006) evaluated the risk factors associated with the increase in the cases of PCOS [82]. On the contrary, a positive correlation was reported between NTx level and the biochemical parameters of glucose, insulin indices, and lipids (*p* < 0.001). Additionally, for PINP serum level, the Pearson coefficient was reported as significant in the case of total testosterone and female testosterone (*p* < 0.05). Metabolic dysfunction, which is associated with obesity, impaired 25(OH)D, and PTH, is critical as it confers a higher risk of PCOS [83].

## 6. Conclusions

Depending on age and clinical diagnosis, PCOS is a multifactorial endocrine and metabolic condition with a higher incidence (up to 20%). Chronic anovulation/oligomenorrhea, hyperandrogenism, and insulin resistance are all clinical signs of PCOS. The biochemical profiles of glucose, lipids, and hormones (PTH, LH, FSH, and 25(OH)D) are among the risk factors linked to PCOS.

The present investigation strengthens and expands the study group’s comprehension of the biochemical and hormonal mechanisms underlying PCOS.

According to the study, the average age of patients with PCOS among obese females was 32 years (20–42 years), and they displayed WCs of 93 cm, BMIs of 29 kg/m^2^, SBPs of 118 mmHg, and DBPs of 82 mmHg. The study also indicates that serum 25 (OH) D levels were considerably decreased in women with PCOS comparison to the control group. The serum NTx levels were found to be greater in women with PCOS in comparison to the control group, indicating the increased impact of PCOS on bone metabolism. The study demonstrated significantly decreased PINP and osteocalcin levels in women with PCOS versus the control group. As bone markers, the Pearson coefficient showed a strong correlation with the biochemical and hormonal variables of NTx, osteocalcin, and PINP. It is evident that the endocrine activity in PCOS females was higher compared to the controls. In the PCOS cases compared to the controls, 25(OH)D and Ca^+2^ levels were decreased. In addition, the serum concentrations of PTH, NTx, and PINP were higher in the PCOS cases compared to the controls, but osteocalcin levels decreased.

In context of the above, the ongoing study arrived at the conclusion that bone formation markers are significantly lower in females with PCOS than in healthy females, perhaps having a long-term effect on these women’s bone mass. Future controlled trials with PCOS women of all ages, such as those who are post menopause and who have never received any type of medication, would allow more precise diagnosis and efficient preventative treatment.

Finally, the research can be repeated with a follow-up of patients for a longer period, especially women who do not respond to treatment, with a follow-up on the impact of the disease on bone health.

## Figures and Tables

**Table 1:** Clinical parameter data of the studied groups.

Variable	Group	N	Mean ± SD	Range		F	P	*p*-Value
**Age (years)**	Group I	100	30.07 ± 5.31	20.00	40.00	1.98	0.06 NS	----
	Group IIa	70	32.67 ± 6.00	20.00	42.00			
	Group IIb	30	30.97 ± 5.64	20.00	39.00			
**WC (cm)**	Group I	100	85.37 ± 8.44	75.00	105.00	39.71	<0.001 **	<0.001 **
	Group IIa	70	93.01 ± 6.27	80.00	105.00			0.004 *
	Group IIb	30	80.50 ± 3.05	75.00	85.00			<0.001 **
**BMI (Kg/m^2^)**	Group I	100	24.76 ± 3.00	20.00	30.00	69.60	<0.001 **	<0.001 **
	Group IIa	70	29.29 ± 3.05	25.00	35.00			0.03 *
	Group IIb	30	23.23 ± 1.65	19.00	25.00			<0.001 **
**SBP (mmHg)**	Group I	100	116.97 ± 5.00	105.00	125.00	3.23	0.06 NS	----
	Group IIa	70	118.89 ± 6.03	100.00	130.00			
	Group IIb	30	116.37 ± 4.63	105.00	122.00			
**DBP (mmHg)**	Group I	100	80.98 ± 4.12	70.00	93.00	1.38	0.25 NS	----
	Group IIa	70	81.54 ± 3.96	73.00	90.00			
	Group IIb	30	80.13 ± 3.10	75.00	88.00			

SD, standard deviation; F, ANOVA test; P1, group I versus group IIa; P2, group I versus group IIb; P3, group IIa versus group IIb; NS, nonsignificant (*p* > 0.05); * significant (*p* < 0.05); ** highly significant (*p* < 0.001).

**Table 2:** Biochemical parameters of glucose and insulin indices among the studied groups.

Variable	Group	N	Mean ± SD	Range		F	P	*p*-Value
**Fasting Glucose (mg/dL)**	Group I	100	87.41 ± 8.13	75.00	103.00	18.26	<0.001 **	0.001 *
	Group IIa	70	94.69 ± 8.52	80.00	113.00			0.01 *
	Group IIb	30	92.10 ± 4.60	87.00	102.00			0.29 NS
**Fasting Insulin (μU/mL)**	Group I	100	13.14 ± 3.66	7.00	15.00	18.74	<0.001 **	0.001 **
	Group IIa	70	16.43 ± 3.06	13.00	32.00			0.02 *
	Group IIb	30	13.17 ± 3.86	11.00	18.00			0.23 NS
**HOMA IR**	Group I	100	2.37 ± 0.73	1.50	2.98	15.23	<0.001 **	0.002 *
	Group IIa	70	3.09 ± 1.04	2.60	8.30			0.005 *
	Group IIb	30	2.94 ± 0.89	2.60	4.00			0.71 NS
**QICKI**	Group I	100	0.34 ± 0.07	0.32	0.36	9.43	<0.001 **	0.001 *
	Group IIa	70	0.29 ± 0.10	0.23	0.31			0.04 *
	Group IIb	30	0.30 ± 0.006	0.28	0.33			0.82 NS

SD, standard deviation; F, ANOVA test; P1, group I versus group IIa; P2, group I versus group IIb; P3, group IIa versus group IIb; NS, nonsignificant (*p* > 0.05); * significant (*p* < 0.05); ** highly significant (*p* < 0.001).

**Table 3:** Biochemical parameters of lipid profiles among the studied groups.

Variable	Group	N	Mean ± SD	Range		F	P	*p*-Value
**Cholesterol (mg/dL)**	Group I	100	165.15 ± 32.6	140.00	170.00	8.77	<0.001 **	0.002 *
	Group IIa	70	189.81 ± 49.01	170.00	210.00			0.81 NS
	Group IIb	30	170.02 ± 25.22	159.00	187.00			0.04 *
**TG** **(mg/dL)**	Group I	100	97.68 ± 5.83	75.00	110.00	1.93	0.15 NS	<0.001 **
	Group IIa	70	99.59 ± 5.43	90.00	110.00			
	Group IIb	30	98.93 ± 6.08	90.00	111.00			
**HDL** **(mg/dL)**	Group I	100	43.69 ± 3.87	35.00	50.00	8.41	<0.001 **	0.01 *
	Group IIa	70	45.93 ± 4.07	35.00	55.00			0.01 *
	Group IIb	30	46.03 ± 3.70	40.00	55.00			0.99 NS
**LDL** **(mg/dL)**	Group I	100	109.46 ± 28.61	78.00	122.00	8.16	<0.001 **	0.003 *
	Group IIa	70	123.13 ± 13.96	95.00	150.00			0.92 NS
	Group IIb	30	111.27 ± 10.07	99.00	125.00			0.04 *
**Non-HDL (mg/dL)**	Group I	100	124.14 ± 28.60	92.00	131.00	12.3	<0.001 **	0.001 *
	Group IIa	70	143.90 ± 30.70	115.00	170.00			0.49 NS
	Group IIb	30	130.20 ± 16.05	120.00	145.00			0.04 *

Sd, standard deviation; F, ANOVA test; P1, group I versus group IIa; P2, group I versus group IIb; P3, group IIa versus group; NS, nonsignificant (*p* > 0.05); * significant (*p* < 0.05); ** highly significant (*p* < 0.001).

**Table 4:** Biochemical parameters of PCOS among the studied groups.

Variable	Group	N	Mean ± SD	Range		F	P	*p*-value
**FSH (U/L)**	Group I	100	6.99 ± 2.33	5.60	10.20	3.02	0.06 NS	-----
	Group IIa	70	6.20 ± 2.10	4.00	8.00			
	Group IIb	30	6.31 ± 1.86	4.80	7.90			
**LH (U/L)**	Group I	100	4.18 ± 0.61	3.00	5.30	737.34	<0.001 **	<0.001 **
	Group IIa	70	8.80 ± 1.07	6.80	11.10			<0.001 **
	Group IIb	30	8.65 ± 0.91	7.30	10.00			0.71 NS
**PRL** **(ng/mL)**	Group I	100	18.05 ± 5.49	12.00	22.00	2.81	0.06 NS	-----
	Group IIa	70	19.89 ± 4.78	15.00	25.00			
	Group IIb	30	19.37 ± 4.55	15.00	25.00			
**Estrogen** **(pg/mL)**	Group I	100	76.88 ± 14.79	50.00	100.00	385.05	<0.001 **	<0.001 **
	Group IIa	70	130.00 ± 13.31	100.00	150.00			<0.001 **
	Group IIb	30	133.93 ± 11.69	112.00	152.00			0.40 NS
**SHGB** **(nmol/L)**	Group I	100	53.10 ± 6.89	30.00	67.00	149.16	<0.001 **	<0.001 **
	Group IIa	70	38.01 ± 6.51	25.00	50.00			<0.001 **
	Group IIb	30	35.53 ± 5.06	28.00	50.00			0.19 N
**Female Total Testosterone** **(ng/dL)**	Group I	100	62.59 ± 20.86	20.00	79.00	10.06	<0.001 **	0.001 *
	Group IIa	70	78.53 ± 26.83	65.00	92.00			0.04 *
	Group IIb	30	74.33 ± 23.79	59.00	80.00			0.51 N
**Female FREE Testosterone (ng/dL)**	Group I	100	1.18 ± 0.06	0.66	1.56	6.99	0.001 *	0.001 *
	Group IIa	70	1.33 ± 0.44	1.02	1.81			0.04 *
	Group IIb	30	1.32 ± 0.23	1.03	1.60			0.99 NS
**Female Testosterone (%)**	Group I	100	1.43 ± 0.43	1.11	1.90	8.11	<0.001 **	0.005 *
	Group IIa	70	1.69 ± 0.48	1.40	2.13			0.04 *
	Group IIb	30	1.65 ± 0.35	1.40	2.00			0.91 N
**DHEAS (μg/dL)**	Group I	100	45.48 ± 6.91	34.00	60.00	66.65	<0.001 **	<0.001 *
	Group IIa	70	60.33 ± 10.53	45.00	79.00			<0.001 **
	Group IIb	30	55.97 ± 7.79	45.00	72.00			0.05 *

Sd, standard deviation; F, ANOVA test; P1, group I versus group IIa; P2, group I versus group IIb; P3, group IIa versus group IIb; NS, nonsignificant (*p* > 0.05); * significant (*p* < 0.05); ** highly significant (*p* < 0.001).

**Table 5:** Bone turnover markers among the studied groups.

Variable	Group	N	Mean ± SD	Range		F	P	*p*-Value
**Parathyroid Hormone** **(pg/mL)**	Group I	100	57.70 ± 15.54	30.00	50.00	1.8	0.17 NS	-----
	Group IIa	70	62.33 ± 14.95	55.00	70.00			
	Group IIb	30	61.43 ± 14.46	55.00	69.00			
**Ca (mg/dL)**	Group I	100	9.18 ± 1.45	8.50	9.90	6.05	0.002 *	0.008 *
	Group IIa	70	8.68 ± 0.40	7.30	9.20			0.03 *
	Group IIb	30	8.61 ± 0.34	8.00	9.40			0.95 NS
**Ph (mg/dL)**	Group I	100	3.13 ± 1.04	2.40	4.00	9.85	<0.00 **	0.001 *
	Group IIa	70	3.9 ± 1.33	3.50	4.90			0.02 *
	Group IIb	30	3.80 ± 1.27	3.40	4.20			0.92 NS
**ALP (U/L)**	Group I	100	81.22 ± 14.27	55.00	105.00	2.26	0.11 NS	----
	Group IIa	70	85.34 ± 10.54	60.00	105.00			
	Group IIb	30	82.13 ± 10.80	62.00	100.00			
25(OH)D **(ng/mL)**	Group I	100	30.34 ± 5.67	20.00	40.00	47.10	<0.001 **	<0.001 **
	Group IIa	70	22.96 ± 4.15	14.00	29.00			0.002 *
	Group IIb	30	26.83 ± 3.37	20.00	35.00			0.001 *
**NTx** **(nM BCE/mM creatinine)**	Group I	100	34.07 ± 7.87	20.00	50.00	2.48	0.09 NS	-----
	Group IIa	70	33.96 ± 6.22	24.00	50.00			
	Group IIb	30	37.20 ± 6.98	25.00	50.00			
**Osteocalcin (ng/mL)**	Group I	100	20.17 ± 3.34	15.00	25.00	195.01	<0.001 **	<0.001 **
	Group IIa	70	11.46 ± 2.99	5.00	20.00			<0.001 **
	Group IIb	30	11.67 ± 2.44	6.00	15.00			0.95 NS
**PINP (μg/L)**	Group I	100	57.80 ± 11.75	40.00	80.00	145.97	<0.001 **	<0.001 **
	Group IIa	70	35.97 ± 6.42	25.00	45.00			<0.001 **
	Group IIb	30	33.43 ± 5.21	25.00	40.00			0.43 NS
**BMD T-score**	Group I	100	0.41	−0.65	0.78	4.38	0.13 NS	----
	Group IIa	70	0.29	−1.56	0.78			
	Group IIb	30	0.23	−1.50	0.70			
**BMD Z-score**	Group I	100	0.36	−0.42	0.93	4.71	0.10 NS	----

Sd, standard deviation; F, ANOVA test; P1, group I versus group IIa; P2, group I versus group IIb; P3, group IIa versus group IIb; NS, nonsignificant (*p* > 0.05); * significant (*p* < 0.05); ** highly significant (*p* < 0.001).

**Table 6:** Correlation between bone markers and different parameters among cases groups.

Parameters and Groups		NTx (nM BCE/mM Creatinine)	Osteocalcin (ng/mL)	PINP (μg/L)
Osteocalcin (ng/mL)	r	0.030	---	−0.046
	P	0.768	---	0.647
PINP (μg/L)	r	−0.135	−0.046	---
	P	0.181	0.647	---
Age (years)	r	0.047	0.188	0.059
	P	0.644	0.061	0.563
WC (cm)	r	−0.136	0.061	0.200*
	P	0.176	0.544	0.046
BMI (Kg/m^2^)	r	−0.250 *	0.018	0.165
	P	0.012	0.859	0.101
SBP (mmHg)	r	−0.010	0.139	0.054
	P	0.920	0.167	0.592
DBP (mmHg)	r	−0.070	0.195	0.058
	P	0.491	0.052	0.567
Fasting Glucose (mg/dL)	r	−0.094	0.015	−0.196
	P	0.354	0.886	0.051
Fasting Insulin (μU/mL)	r	−0.352 **	0.006	0.143
	P	<0.001	0.955	0.156
HOMA IR	r	−0.343 **	−0.004	0.046
	P	<0.001	0.970	0.646
QICKI	r	0.348 **	−0.021	−0.125
	P	<0.001	0.833	0.216
Cholesterol (mg/dL)	r	−0.197 *	−0.018	−0.036
	P	0.049	0.863	0.723
TG (mg/dL)	r	0.005	0.080	−0.066
	P	0.964	0.428	0.514
HDL (mg/dL)	r	0.038	0.104	−0.120
	P	0.708	0.302	0.236
LDL (mg/dL)	r	−0.166	−0.094	0.001
	P	0.098	0.353	0.993
Non-HDL (mg/dL)	r	−0.188	−0.051	0.010
	P	0.061	0.613	0.920
FSH (U/L)	r	−0.022	0.047	−0.077
	P	0.828	0.645	0.449
LH (U/L)	r	−0.094	−0.050	−0.024
	P	0.352	0.624	0.809
PRL (ng/mL)	r	0.001	−0.294 *	0.083
	P	0.990	0.003	0.412
Estrogen (pg/mL)	r	0.138	0.068	0.049
	P	0.172	0.502	0.631
SHGB (nmol/L)	r	0.110	0.117	−0.144
	P	0.278	0.247	0.153
Total Testosterone (ng/dL)	r	−0.187	0.027	0.201 *
	P	0.062	0.787	0.044
Female Testosterone (ng/dL)	r	−0.215 *	−0.069	0.249 *
	P	0.032	0.492	0.013
Female Testosterone (%)	r	−0.124	−0.132	0.159
	P	0.219	0.191	0.115
DHEAS (μg/dL)	r	−0.031	0.089	0.148
	P	0.763	0.378	0.141
Parathyroid Hormone (pg/mL)	r	0.047	0.256 *	0.161
	P	0.643	0.010	0.109
Ca (mg/dL)	r	0.131	−0.099	−0.162
	P	0.194	0.325	0.108
Ph (mg/dL)	r	−0.134	0.158	0.180
	P	0.182	0.116	0.073
ALP (U/L)	r	0.076	0.278 *	0.100
	P	0.451	0.005	0.321
25(OH)D (ng/mL)	r	0.140	0.218 *	−0.156
	P	0.164	0.029	0.121

r, Pearson’s correlation coefficient; NS, non-significant (*p* > 0.05); * significant (*p* < 0.05); ** highly significant (*p* < 0.001).
